# **Prospects of Maize** **(Corn) Wet Milling By-Products as a Source of Functional Food Ingredients and Nutraceuticals**

**DOI:** 10.17113/ftb.60.01.22.7340

**Published:** 2022-03

**Authors:** Thalli Satyanarayana Deepak, Padmanabhan Appukuttan Jayadeep

**Affiliations:** 1 Grain Science and Technology Department, CSIR-Central Food Technological Research Institute, Mysore, India; 2 Academy of Scientific and Innovative Research, Ghaziabad, India

**Keywords:** maize (corn), wet milling, by-products, nutraceuticals, functional food ingredients

## Abstract

Maize (corn) consists of germ, endosperm and pericarp, with different chemical compositions. During wet milling, the maize is disintegrated into the main product starch and by-products, including maize germ, maize fibre and maize gluten (the technical term for maize/corn endosperm-specific proteins and not the same as wheat gluten). These by-products are used as low-value animal feed products. The maize germ contains high amounts of tocols and phospholipids, the maize gluten is rich in carotenoids and the maize fibre fraction is rich in phytosterols and complex carbohydrates. Each by-product has a potential to serve as a precursor in the manufacture of functional food ingredients or nutraceuticals that have antioxidant, anti-inflammatory, hypocholesterolaemic, hypolipidaemic and hypoglycaemic properties. These food ingredients/nutraceuticals can be obtained through physical, chemical or enzymatic processes. Some nutraceuticals and food ingredients with market potential include maize/corn fibre gum, oil, arabinoxylans and xylooligosaccharides from maize fibre; maize germ oil and phospholipid ester from maize germ; and carotenoids and oligopeptides from maize gluten. This review focuses on current and prospective research into the use of maize germ, maize fibre and maize gluten in the production of potentially high-quality food ingredients or nutraceuticals.

## INTRODUCTION

Maize (*Zea mays* L.), which belongs to the family *Gramineae* and genus *Zea*, is a staple food in many places worldwide and the third most important crop after rice and wheat (*1*). World maize production is currently 1136 million metric tonnes (MMT) and in India, it is 30.2 MMT (*2*). Due to its diverse uses as a food crop, animal feed and important raw material, maize is a crop that is of vital importance around the world. It is used to manufacture various food and industrial products such as starch, animal feed, sweeteners, beverages, oil, glue, industrial alcohol and ethanol for fuel (*3*).

About 20% of the maize produced in India is used for food purposes, about 47% for poultry feed, 13% for livestock feed, 14% in the wet milling industry to obtain starch and 6% for export and industrial non-food products (*4*). The maize is mainly processed into food using dry and wet milling procedures. The wet milling process mainly concentrates on starch and its derivatives from maize. Starch undergoes various physical, enzymatic or chemical modifications in order to obtain various products. Some products are maltodextrin, dextrin, dextrose monohydrate, sorbitol, liquid glucose, high maltose syrup, dextrose syrup and anhydrous dextrose. These are used to produce beverages, bakery goods, pastries, meat, soups, sauces or baby food, textiles, dextrins, paper and pharmaceutical products (*5*). In addition to starch, the by-products corn steep liquor, maize germ, corn fibre and corn gluten (technical term for corn endosperm-specific proteins) are obtained. This review aims to provide current literature on the various studies of the nutraceutical composition of the by-products and the gap existing therein. It also focuses on the used opportunities and prospects for the valorisation of by-products to produce value-added components, functional food ingredients and nutraceuticals.

### Maize wet milling

Maize wet milling involves the process of various physical, chemical, biochemical and mechanical operations to separate the components of the maize grain (germ, steep liquor, starch and maize gluten) into valuable products that are far more worthy than the raw grain (*6*). Maize starch and maize germ oil are the main profitable products of the maize wet milling industry (*7*). With starch (60- 70%) being the main product, the by-products include steep liquor/solubles, maize germ, maize fibre and maize gluten (*6*).

The corn wet milling industry started in 1844 in the United States of America by Thomas Kingsford of the William Colgate and Company in Jersey City, NJ, using a new alkali process to extract starch from maize (*5*). At the initial stages, the maize industry discarded the maize fibre, maize germ and protein obtained during the processing. Over time, however, the wet milling process gradually changed so the non-starch components have found applications in animal feed, oil, polymer and pharmaceutical industries (*5*).

**Fig. 1:**
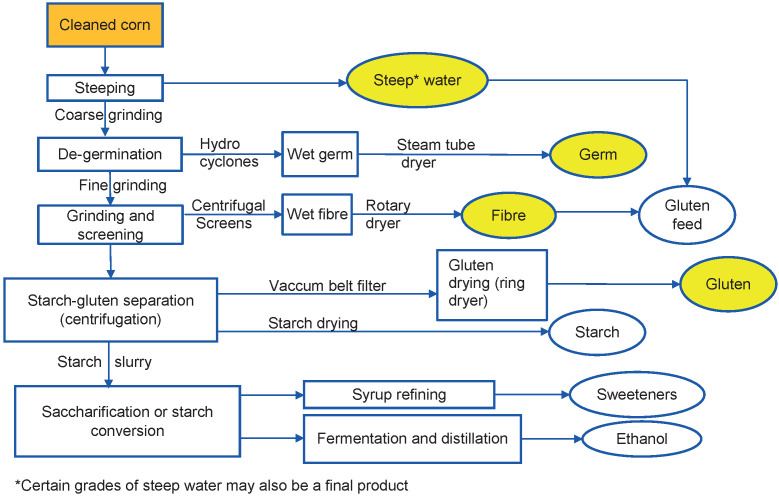
Industrial corn wet milling process (yellow colour represents the main by-products) based on Rausch *et al.* (*6*)

Conventionally, the wet milling process includes grain handling, steeping, separation and product recovery (Fig. 1 (*6*)).

The kernels are cleaned, separated according to their quality and conveyed to steeping tanks. In this process, maize kernels are steeped in SO_2_ (0.2%) and lactic acid (0.5%) solution for 24 to 48 h to facilitate kernel hydration and leaching, and induce mechanical stress for efficient separation of the kernel components (*6*). Initially, lactic acid hydrates the kernels followed by leaching of the soluble materials into the steep water. The soluble carbohydrates of the maize are fermented to produce lactic acid. Later, water uptake results in the swelling and loosening of the connecting cells between the component parts. When SO_2_ starts reacting with the mixture, it restricts fermentation and facilitates starch separation from the protein matrix in the endosperm (*6*). Consequently, the components are then sequentially separated through industrial processes to obtain the end products.

After steeping, the steep water is drained and dried to form condensed steep solubles. Then, the steeped maize kernels are coarsely ground to form a slurry using disk mills with intermeshed teeth to free the maize germ from endosperm and hull. Following this step, the slurry is pumped through a two-stage hydrocyclone system to recover the maize germ, which is further dried. The germ is usually delivered to oil mills, purified and sold for human consumption. Residual maize germ meal is used as a part of livestock feed. Once the germ is separated from the slurry, constituting maize fibre, maize starch and maize gluten, it is finely ground in the plate or single-disk mills. Maize fibre is extracted from the obtained fine slurry by screening and centrifugation, followed by washing and drying. From this defibred slurry, the maize starch and maize gluten are separated based on their differences in densities by the disk-nozzle centrifuge. Subsequent starch washing in hydrocyclones further removes the maize gluten. A mill stream thickener dewaters maize gluten, which is further passed through a vacuum belt filter and then dried by a ring dryer. Both starch and maize gluten are dried separately (*6*, *8*).

Industrially, the starch yield is about 60–70%, maize germ yield is about 7%, maize gluten constitutes 5–6%, maize fibre about 12% and steep water solubles about 7% (*9*). Starch is the primary product, but the by-product value can significantly affect overall plant economics.

#### Current uses of maize wet milling by-products

**Fig. S1:**
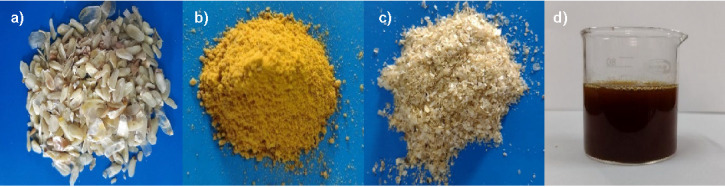
Industrial maize wet milling by-products: a) maize germ, b) maize gluten, c) maize fibre, and d) corn steep liquor

Steep solubles (corn steep liquor) are currently used as a nutrient-rich medium for antibiotic production (*e.g.* penicillin) and as a feed additive for livestock, aquaculture and poultry. Steep solubles are used as feed additive up to 12% of the diet dry matter without adversely affecting feed intake (*10*). Corn steep liquor, also known as condensed fermented maize extractives, is a high protein ingredient. It is often used as a maize gluten feed constituent but may be sold as 50% dry solids for cattle feeds or as a pellet binder (*10*). In some cases, maize germ is used directly to feed ruminants like Holstein cows or mid-lactation dairy cows. Maize gluten feed is a combination of the hulls and fibre fraction with steep water, maize germ meal and other process residuals. Maize gluten meal is the concentrated and dried maize protein obtained after the final separation from starch (*11*). The gluten/gluten meal is a rich protein source and also contains carotenoids. Maize gluten meal is the main feed for poultry and fish to achieve better pigmentation of egg and flesh. The maize fibre is recombined with steep liquor and used as poultry feed. The maize germ meal, maize fibre and steep solubles are recombined to produce the co-product called maize gluten feed used as animal feed (*11*). The industrial maize wet milling by-products are shown in Fig. S1.

By-products account for 30–40% of the total product yield, but 20−25% of the kernel is processed without added value, even though maize germ oil and maize gluten meal have a higher value than starch in the US market (*6*). A wide variety of products can be made from wet-milled starch and by-products, while the use of the by-products in human food is limited.

The use of maize wet milling is increasing, mainly to obtain compounds used in industrial ethanol production. Biofuel (ethanol) production from corn in the US and Brazil and the conversion of food into fuel is a cause for concern (*12*). There will be a justification for the biofuel industry if it adds value to by-products for functional foods. Bioethanol production from corn in the US is growing rapidly as an alternative to increasing gasoline prices and the national renewable fuel program (*13*). Due to increased bioethanol production by maize wet milling, the volume of the by-products has also grown substantially, creating a need for the ethanol industry to find new uses for these by-products and an opportunity for the food ingredient and nutraceutical manufacturers (*13*).

Adequate research and commercial interest in improving the existing by-products can help identify new industrial uses or nutraceuticals from by-products (*6*). Traditionally these by-products of maize wet milling are mainly used as animal feed, which has lower value. Therefore, specific research is needed to study and improve the use of these by-products. Nutraceuticals and functional food ingredients are highly valued, profitable and offer economic relief to farmers by increasing income. It would benefit the wet milling industry and consumers by reducing fuel and food costs.

## PHYTOCHEMICAL COMPONENTS OF MAIZE WET MILLING BY-PRODUCTS

### Maize germ and its co-products

The maize germ makes up 9–11% of the kernel mass, 80−84% of the total kernel oil is located in the maize germ, 12% in the aleurone layer and 5% in the endosperm (*9*). Characterized by a 39–47% lipid content, the maize germ has an 18−19% protein content and starch content of 8% (*9*, *14*). The health-beneficial phytochemicals present in maize germ are antioxidant tocols, cholesterol-lowering phospholipids, phytosterols and policosanols (*15*). Weber (*16*) found 63–91% of tocols in the maize germ and tip cap, 3−11% in the floury endosperm and 6–26% in the horny endosperm. In the case of dry milling, the obtained germ contains 46.3 mg/100 g of total tocols (*17*). The phospholipid content in the maize germ of dissected kernels of high amylose maize (amylomaize), LG-11 hybrid maize and waxy maize was 1224, 1453 and 1363 mg/100 g, respectively (*14*). The serum lipid-reducing policosanol content in the maize germ was 1.93–3.71 mg/100 g (*15*).

The wet-milled maize germ was reported to have 11–16% protein and more than 30% oil (*18*). The maize germ, derived from lab-scale wet milling, had a total tocol content of 8.5 mg/100 g, including 6.6 mg/100 g of γ-tocopherol and 1.9 mg/100 g of α-tocopherol. However, the tocotrienol content was not present in measurable amounts (*19*). At 16.8 mg/100 g, the phytosterol content in wet-milled maize germ was higher than in wet-milled sorghum germ (*20*).

#### Maize germ oil

Maize germ oil is the product of the mechanical expulsion/solvent extraction of oil from maize germ in the wet milling industry. The significant presence of phytosterol in maize germ oil makes it suitable for reducing cholesterol absorption. The phytosterols limit the absorption of cholesterol mainly in the intestinal area and thus effectively prevent cardiovascular diseases (*15*). The tocols (tocopherols and tocotrienols) are dietary antioxidants in maize germ oil which enhance its oxidative stability. They also protect against cell damage caused by free radicals and are helpful in maintaining food quality and health. Phenolic acids are about three times higher in cold-pressed maize germ oil than refined maize oil and can be used as functional ingredients (*21*). It is considered beneficial to health mainly due to its polyunsaturated fatty acids: oleic and linoleic acids. A high oil content (80−84%) in hybrids can improve the commercial prospects of maize germ oil (*9*).

According to a previous study (*18*), the tocopherol content of oil extracted by Soxhlet extraction using *n*-hexane from unroasted wet-milled maize germ was 59.4 mg/100 g. The oil also had a high  γ-tocopherol content of 41.9 mg/100 g, followed by 7.92 mg/100 g of β-tocopherol, 6.26 mg/100 g of α-tocopherol and 3.23 mg/100 g of δ-tocopherol (*18*).

The phytosterol content of the same maize germ oil mentioned above was 713.9 mg/100 g with 504.64 mg/100 g sitosterol, 155.8 mg/100 g campesterol and 53.3 mg/100 g stigmasterol (*18*). In a comparative study (*22*), the tocopherol content in maize germ oil was around 28 times higher than in maize fibre oil.

#### Defatted maize germ flour

Defatted maize germ flour can be a nutritious component of food. In this context, flour was prepared from the wet-milled maize germ containing 30% protein with 5.9% lysine and a balanced ratio of other essential amino acids. Dried maize germs were first aspirated to remove the hull and flake it. The lipid was removed by solvent extraction and the final product contained significantly low lipid levels by a second solvent extraction of the flakes with 82:18 hexane/ethanol azeotrope *via* refluxing. The maize germ flakes were then ground in a grinder. The analysis showed that ground flour contained 2% ash, 18% starch and 0.6% lignin. The flour contained high amounts of dietary fibre (22–29% pentosans and 11–13% cellulose) (*23*). Defatted protein-rich maize germ flour can be a functional ingredient that is added with other types of flour to make chapatis or bread.

#### Phospholipids as a functional ingredient

Maize germ is rich in amphiphilic molecules called phospholipids (*14*). They have hydrophobic fatty acid chains and hydrophilic units that occur in the cell membrane. They are therefore mainly found in all animal- and plant-based foods.

The residual maize germ meal left after the maize germ oil extraction is usually discarded but is rich in lecithin. Lecithin is a high-quality additive component, which exhibits advantageous interfacial properties and has aroused increasing interest as a natural emulsifier in the food, pharmaceutical and cosmetic industries (*24*). It comprises a concentrated mixture of phospholipids such as phosphatidylcholine, phosphatidylethanolamine, phosphatidylinositol, phosphatidic acid and phosphatidylserine (*24*). Lecithin is usually extracted through multi-stage solvent extraction (*24*). Phosphatidylcholine, phosphatidylethanolamine, phosphatidylinositol and phosphatidylserine are the significant phospholipids in maize that regulate brain function and are essential for cell membrane function (*15*). Phospholipids reduce lipid levels in the liver by disrupting sterol absorption in the intestinal cavity. The other phospholipid functions include stimulation of bile acid and cholesterol secretion. Phosphatidylinositol and serine reduce blood triglycerides, fatty liver, bipolar disorders and neurodegenerative diseases (*15*). Since maize germs are rich in phospholipids, they are a potential source for their development as a functional ingredient.

### Maize fibre and its co-products

Maize fibre is a by-product with a second high yield after starch in the maize wet milling process. It is commonly known as ‘white fibre’, a mixture of fibres of the pericarp (bran) and hull (coarse fibre) and fibre of the maize germ cell wall and endosperm (fine fibre) (*25*). The wet-milled maize fibre consists of complex carbohydrates (non-starchy polysaccharides), composed of 40% hemicellulose, 25% starch, 12% cellulose, 10% protein, 3% oil and 10% other substances such as phenolic antioxidants, ferulate phytosterol esters, lignin and ash (*26*). The hemicelluloses (arabinoxylans and β-glucans) are reported to have antioxidant activity (*27*, *28*). Phytochemicals, mainly phytosterols and tocopherols, are also present in maize fibre. Hexane extract of maize fibre was found to contain a phytosterol mass fraction of 19.3 mg/100 g, which was the highest among the wet-milled by-products compared to maize germ (16.8 mg/100 g) and maize gluten (4.8 mg/100 g) (*20*). The tocols were extracted using ethanol from wet-milled maize fibre (lab-scale) and the total tocol levels were observed to be 5.4 mg/100 g (*19*). The homologues, γ-tocopherol (1.47 mg/100 g), α-tocopherol (0.73 mg/100 g), α-tocotrienol (1.29 mg/100 g) and γ-tocotrienol (1.98 mg/100 g) were found in the lab-scale wet-milled maize fibre fraction (*19*).

Dietary fibre consists of insoluble non-starchy polysaccharides present in maize fibre that helps the physiological processes in the grain (*29*). The total dietary fibre mass fraction was 52.6–73.5 g/100 g in wet-milled maize fibre (*30*). Based on previous studies, maize fibre is a potential source of arabinoxylans, a typical dietary fibre (*25*). The soluble fibre components of maize are arabinoxylans, fundamentally situated in pericarp cell walls, where they support the kernel structurally and functionally. These arabinoxylans have arabinose side chains esterified with phenolics like ferulic acid and exert antioxidant properties (*29*).

#### Maize/corn fibre gum

Corn fibre gum (CFG) is an alkaline extract of maize fibre comprised of arabinoxylan (hemicellulose) with high solubility and low viscosity (*7*, *25*). CFG contains less than 5% proteins, phenolics (ferulic and *p*-coumaric acids) and lipids (*27*). The hemicellulose from maize fibre has properties similar to gum arabic and is often expensive and scarce. Hemicellulose is extracted with hot alkali, NaOH/Ca(OH)_2_ and bleached with hydrogen peroxide. CFG extracts were obtained as both acid-soluble and acid-insoluble fractions (*25*). CFG is an excellent emulsifier with improved physicochemical and nutritional properties due to the presence of phenolic acids, lipids and proteins (*7*). Additionally, it can be an adhesive, thickener, stabilizer and film former. Therefore, the viability of CFG application in expanded snacks, bakery goods, beverages, specialty foods, edible coatings, nutritional supplements and development of functional foods due to its health benefits can be studied.

Dietary fibre is the main constituent of maize fibre and it assists in the proper functioning of the digestive system by acting as a stool bulking agent (*29*). Arabinoxylans (both soluble and insoluble) can improve colon function, prevent diabetes mellitus, cardiovascular diseases, some cancers and immunological disorders (*7*). They have a robust prebiotic effect, reduce gut infections, prevent colon cancer and increase the amount of intestinal short-chain fatty acids known to reduce blood cholesterol (*7*, *29*). Arabinoxylans have been used to produce functional gluten-free bread with improved properties (*7*).

#### Xylooligosaccharides

Xylooligosaccharides (XOS) are potential prebiotics, partially hydrolyzed, water-soluble xylan fragments obtained from maize fibre by enzymatic or high-temperature treatment (*7*). Microbial xylanases are used for the enzymatic degradation of maize fibre. In contrast, hydronium ions and organic acids are used for high-temperature treatment at 160–220 °C to partially hydrolyze heteroxylan polymers and yield soluble hydrolysates of XOS (*31*). Preliminary studies of the use of XOS structurally similar to maize fibre in the diet of older Japanese men showed an increase in the number of bifidobacteria after three weeks of their consumption (*32*). In addition to its prebiotic activity, the XOS may exhibit antioxidant activity due to the bonding of ferulic acid ester moieties to the solubilized oligosaccharides (*7*).

#### Ferulic acid

Ferulic acid has many physiological functions, including antioxidant, antimicrobial, anti-inflammatory, antithrombotic and anticancer activities (*33*). Due to its antioxidant and antimicrobial properties, ferulic acid can be an excellent preservative. An expensive chemical process is used to produce commercial ferulic acid with environmental concerns, and therefore there is a need for natural sources to obtain this product. Maize bran contains good amounts of ferulic acid (30 g/kg) compared to rice bran oil (10–20 g/kg) and can therefore be an alternative source of ferulic acid (*7*). Unfortunately, ferulic acid in maize bran is bound to the cell wall, thus posing major challenges in its isolation. Experiments on the extraction of bound ferulic acid from maize bran have been conducted. After a more aggressive alkali (NaOH) treatment of maize bran to break the ferulate crosslinks, a high amount of ferulic acid was released into the solution (*34*). As a result, the corn fibre gum (CFG) and ferulic acid are co-solubilized and the ferulic acid is separated by precipitating CFG in ethanol (*34*). Another study demonstrated that thermal and enzymatic treatments release ferulic acid from maize bran (*35*). Obtaining ferulic acid from maize fibre is an attractive opportunity to transform the maize wet milling industry.

#### Xylitol

Xylitol is a naturally occurring five-carbon sugar alcohol derived from xylose by reduction of the carbonyl group. It is used as a low-calorie sweetener in the food industry as it is helpful for people with diabetes and has bulking properties similar to sucrose (*7*). It does not cause tooth decay, it is slowly absorbed and has a lower glycaemic index (*36*). The chemical hydrogenation of xylose produces the major part of xylitol, usually obtained from the wood hydrolysate. Maize fibre can be a potential starting material as it has a xylose mass fraction (approx. 200 g/kg) similar to that of hardwood (*7*).

In an experiment employing yeast, xylose is initially hydrolyzed with dilute acid or enzyme treatment in a cost-effective step. The resulting syrup is then fermented by a yeast *Candida tropicalis* and treated with activated charcoal to remove xylitol inhibitors and increase xylitol yield (*37*, *38*).

#### Vanillin

Vanillin is an essential flavour and aroma compound used in the food, pharmaceutical and cosmetic industries. It is obtained from properly cured vanilla (*Vanilla planifolia* Andrews) pods with a mass fraction of 10–30 g/kg. Vanillin is also found in agricultural products, including pine, tobacco and citrus fruits (*7*). In maize bran, vanillin mass fraction is about 55 g/100 g (*39*). The demand for vanillin exceeds its production from vanilla beans. Many researchers have turned to the microbiological conversion of ferulic acid to vanillin (*40*).

A procedure involving *Aspergillus niger* was used to produce vanillin from ferulic acid obtained from autoclaved maize bran (*40*). Recently, one process used pressurized subcritical water (low polarity water) to convert ferulic acid to vanillin by breaking its aliphatic double bond (*39*). It is also known that maize fibre contains sufficient amounts of ferulic acid and, therefore, can be used for vanillin production.

#### Maize fibre oil

Maize fibre oil is a unique oil extracted from finely ground maize fibre, usually with hexane, ethanol or supercritical CO_2_ (*41*)_._ The total phytosterol content in maize fibre oil is estimated to 7939 mg/100 g compared to 840 mg/100 g of maize germ oil (*42*), but the yield of maize fibre oil obtained with hexane is about 2–3% (*41*). The maize fibre contains starch, hemicellulose and cellulose, which must be removed to improve maize fibre oil yield. After treatment with dilute acids and enzymes, the phytosterol mass fraction in maize fibre increased from 19.8 to 1256.2 mg/g (*26*).

Maize fibre oil has recently been of interest due to high content of ferulate phytosterol esters, the most predominant being sitostanyl ferulate (*42*, *43*). Additional animal studies have confirmed its cholesterol-lowering effects (*43*, *44*).

#### Policosanols

Policosanols are a mixture of long-chain primary aliphatic alcohols mainly abundantly found in sugarcane. These compounds were identified and first approved as dietary supplements in Cuba and are commercialized in the Caribbean and South American countries (*45*). As mentioned earlier, the pericarp fraction of the wet-milled maize fibre contains policosanols. The maize pericarp has a policosanol content of 72.7–110.9 mg/kg (*46*). Physiologically they improve health by reducing blood lipid levels and platelet aggregation (*15*). Octacosanol (C28), triacontanol (C30) and hexacosanol (C26) are primarily reported policosanols that contribute to the lowering of serum cholesterol levels. The distillers' dried grains with solubles obtained after fermentation of dry milled maize by-products contain policosanols and can be converted into health-promoting functional ingredients (*15*).

Several dietary supplements are commercially available in the US market containing policosanol, usually derived from sugarcane (*15*, *46*). Numerous scientific studies indicate that the daily consumption of 1–20 mg of policosanols effectively reduces insulin resistance, total blood cholesterol and LDL-cholesterol in older adults (>75 years) (*15*). Further research is needed to determine the policosanol components in maize fibre.

### Maize gluten (proteins) and its co-products

Maize gluten meal (MGM) (corn protein) is a significant maize wet milling by-product containing at least 60% protein and is rich in health-promoting carotenoids (*10*). Maize protein is not the same as wheat gluten which causes coeliac disease. Maize contains albumins, globulins, prolamines (zein protein) and glutelin proteins (35%), with zein protein contributing more than 50%. A mixture of zein protein and glutelins, known industrially as maize gluten, are endosperm-specific (*47*). MGM contains adequate quantities of sulfur-containing amino acids, methionine and cysteine, involved in synthesizing intracellular antioxidants (*10*). The hydrophobic amino acid composition containing leucine, alanine and phenylalanine makes MGM proteins a good source of bioactive peptides (*48*). However, due to its imbalanced amino acid composition and low water solubility proteins, MGM is mainly marketed as a feedstock or discarded but not used for human food production (*49*). Nevertheless, maize gluten meal hydrolysis can provide peptides with antioxidant properties and can therefore be revalorized in food or pharmaceutical products (*48*, *49*).

Low phytosterol mass fractions of 4.8 mg/100 g were detected in the wet-milled gluten (*20*). Phytochemically, yellow maize contains 74–86% carotenoids (primarily xanthophylls) in the endosperm trapped in the gluten matrix (*1*). The MGM contained around 195–491 mg/kg xanthophylls (lutein, zeaxanthin and cryptoxanthin), whereas the feed maize kernels had only 40.1 mg/kg xanthophylls (*10*, *50*). The carotene mass fraction of MGM was 49–73 mg/kg, while it was 22 mg/kg in the feed maize material (*10*). These studies indicate that xanthophylls and carotenes are concentrated in the MGM obtained from the maize wet milling. Zeaxanthin is a significant component in cooked maize compared to other foods such as spinach, lettuce and parsley (*51*). MGM is characterized by high protein and energy content making it a potential high energy source of nutrition.

#### Carotenoid supplements

Functional carotenoids can be extracted from MGM by solvent extraction (hexane, acetone or ethanol) assisted with mechanical/physical methods (maceration, microwave or ultrasound), supercritical fluid (CO_2_) extraction or Soxhlet extraction (*52*). Carotenoids are excellent natural antioxidants used for maintaining food quality and human health (*15*). They improve eye health, prevent cancer and have anti-aging properties. Lutein and zeaxanthin are the carotenoids in the macula of the retina necessary for sharp and detailed vision. Studies have shown that they protect the eye from phototoxic damage, age-related macular degeneration and cataract formation (*53*). Lutein also inhibits cancer as a chemopreventive and suppressive agent (*54*). Extraction and utilization of carotenoids, mainly lutein and zeaxanthin, from maize gluten as dietary supplements or food ingredients is an exciting prospect as it is a rich and cheap source.

#### Maize protein hydrolysates and bioactive peptides

Maize gluten meal (MGM) contains diverse proteins, including albumins, globulins, glutelins and prolamins (zein protein). It is reported that the bioavailability of proteins of MGM can be remarkably enhanced by enzymatic hydrolysis. As a result, hydrolysates are obtained containing small peptides, especially dipeptides and tripeptides, that can be absorbed more efficiently than the intact proteins or the free amino acids (*49*). These protein hydrolysates are antioxidative and can effectively inhibit lipid oxidation in foods (*55*). Additionally, they can be used as food additives and for the preparation of edible coatings and packaging films.

Bioactive peptides are encoded specific peptide fragments within the primary structure of proteins that remain inactive and have potential health benefits (*56*). After the protein hydrolysis, bioactive peptides are released that can modulate human metabolism, and also treat chronic diseases with discrete potency and fewer side effects such as toxicity. Enzymatic hydrolysis is predominantly used for the production of protein hydrolysates. Alcalase is used, for example, to produce bioactive peptides (*57*). Additionally, the integrated utilization of multiple enzymes or enzymes linked to other techniques was standardized for use in the hydrolysis of MGM (*58*). After hydrolysis, the isolation and purification procedure involves membrane separation (ultrafiltration or nanofiltration) or column chromatography (fast protein liquid chromatography, size-exclusion, and others). The obtained peptides are later characterized by sodium dodecyl sulfate-polyacrylamide gel electrophoresis, mid infrared spectroscopy or mass spectrometry techniques (*59*).

Recently, corn peptides, a novel food derived from MGM through enzymatic hydrolysis or microbial fermentation, have been recognized for their various bioactive properties, including antioxidant activity (*49*, *60*), improvements in lipid profiles and the ability to accelerate alcohol metabolism and protect against alcohol-induced liver injury (*61*). Maize peptides are distinguished as small in size, readily absorbable and safe for consumption. They have been reported to have many inherent bioactive properties such as anti-inflammatory, antioxidant, antihypertensive, hepatoprotective, alcohol metabolism-facilitating, anticancer, antimicrobial and DPP-IV (dipeptidyl-peptidase IV, EC 3.4.14.5) inhibitory activities (*48*, *49*, *58*, *60*). Maize peptides from maize gluten meal show antihypertensive activity with strong ACE (angiotensin-converting enzyme) inhibitory activity (*62*). Many studies including a study on obese rats have examined the anti-obesity effects of maize protein hydrolysates and enriched peptides (*49*). In addition to these properties, some maize peptides also have antimicrobial or metal-binding activities (*63*). Antimicrobial peptides could find exciting applications in the field of food safety. For example, laboratory tests have shown that they protect fresh meat by inhibiting bacterial growth and blocking bacteria from adhering to meat surfaces (*64*).

Phytochemicals from the by-products mentioned above are usually associated with antioxidant and antiradical activities, antimutagenesis, anticarcinogenesis, antimicrobial, anti-inflammatory activities, antilipidaemic and hypocholesterolaemic properties. These phytochemicals may be partially degraded during storage, milling and processing. These aspects have to be examined to study the effects of processing on the nutraceutical quality of the food ingredients. Nevertheless, the maize wet milling by-products are rich in nutraceuticals. Nutraceuticals are nutritional supplements containing concentrated bioactive components from a specific food, incorporated into a non-food matrix, that promote health, applied in dosages that may exceed those obtained from regular food (*65*). Table 1 (*9*, *10*, *12*, *19*, *20*, *30*, *50*, *66*-*73*) shows the phytochemical/nutraceutical composition of maize wet milling by-products. It can be seen that the studies on phytonutrients in maize wet milling by-products are scarce and therefore worth investigating.

**Table 1:** Proximate and phytochemical composition of maize wet milling by-products

**Nutrient**	Maize	Gluten meal	Germ	Fibre	Reference
***w*(protein)/%**	7–13	60–75	20–30	10–13	(*9*, *10*, *50*, *66*–*68*)
***w*(starch)/%**	67–73	12–20	19–25.4	15–20	(*9*, *10*, *50*, *66*–*69*)
***φ*(oil)/%**	2–6	1–6.5	1–20^a^, 40–50^b^	1.72–3.68	(*9*, *10*, *50*, *66*, *70*, *71*)
***w*(ash)/%**	1.4	1.1–4.6	1.6–4.3	6–20	(10,12,50,66–68)
***w*(dietary fibre)/%**	12.19–12.80	4.65	NR	52.6–73.5	(*30*, *69*, *72*)
***w*(phenolics as GAE)/(mg/100 g)**	239.2–327.7	NR	NR	NR	(*73*)
***w*(xanthophyll)/(mg/100 g)**	4.01	19.5–49.1	NR	NR	(*10*, *50*)
***w*(carotene)/(mg/100 g)**	2.2	4.9–7.3	NR	NR	(*10*)
***w*(tocol)/(mg/100 g)**	6.69	7.85	8.5	5.46	(*19*, *73*)
***w*(phytosterol)/(mg/100 g)**	88.01	4.8	16.8	19.3	(*20*)

NR=not reported, ^a^oil in germ meal, ^b^oil in germ, GAE=gallic acid equivalent

The bioactive components are intrinsic to by-products: maize germ is rich in phytosterols and tocopherols, maize gluten is rich in carotenoids and proteins, and maize fibre is rich in phytosterols and dietary fibre, as mentioned above.

A nutraceutical has a physiological benefit or protects against chronic diseases. Nutraceuticals have demonstrated beneficial effects in combating oxidative stress, chronic diseases and cancer.

Some functional food ingredients containing nutraceutical compounds have been developed from maize wet milling by-products. Table 2 (*10*, *14*, *15*, *20*, *22*, *27*, *42*, *43*, *45*, *46*, *49*, *53*, *54*, *60*, *62*, *74*-*80*) shows the nutraceuticals and functional food ingredients from maize and their beneficial effects.

**Table 2:** Health benefits of maize wet milling by-products rich in phytochemicals, and their functional food ingredients

**Phytochemical**	Health benefit	By-product and (co-product) rich in phytochemicals	Functional food ingredient from the by-products	Reference
**Phytosterols: sterols and stanols (*e.g.* β-sitosterol,** **stigmasterol,** **campesterol)**	Reduce cholesterol absorption in the intestine, prevent cardiovascular diseases, reduce oxidized low-density lipoprotein levels, reduce colon tumours, prevent osteoarthritic degradation	Maize bran/fibre (fibre oil) Maize germ (germ oil)	Fibre oil (rich in phytosterols) Cold-pressed maize germ oil	(*15*, *20*, *27*, *42*, *43*, *74*)
**Tocols/tocochromanols (tocopherols and tocotrienols) (α, β, γ, δ)**	Protect cells from free radicals, strengthen the immune system by T-lymphocytes, prevent cardiovascular diseases	Maize germ (germ oil) Maize fibre (fibre oil)	Germ oil Rich in γ-tocopherols Fibre oil	(*15*, *22*, *75*)
**Carotenoids: xanthophylls (lutein, zeaxanthin), β-carotene**	Prevent cancer, protect eye health, prevent cardiovascular diseases and strengthen the immune system	Maize gluten meal	Not available	(*10*, *53*, *54*)
**Policosanols: octacosanol, triacontanol, hexacosanol,** **dotriacontanol**	Reduce blood lipid levels and platelet aggregation	Maize fibre	Not available	(*45*, *46*, *76*)
**Phospholipids: phosphatidyl** **choline, phosphatidyl** **ethanolamine, phosphatidyl inositol, phosphatidyl serine**	Hypocholesterolaemic, cardioprotective, hepatoprotective, hypolipidaemic and anticarcinogenic. Phosphatidyl choline is beneficial in brain and mental development, by neural transmission and can treat neurological disorders	Maize germ	Phospholipid-based emulsifiers (lecithin) from maize are on the market, patented by Cargill company (*e.g.* Nestle-NAN)	(*14*, *15*, *75*)
**Phenolic compounds** **Simple phenolics (ferulic acid)**	Potent antioxidants that prevent inflammation. Ferulic acid bound to soluble corn fibre gum is delivered to the colon to prevent colon inflammatory diseases	Maize fibre	Corn fibre gum, xylooligosaccharides	(*27*, *77*)
**Arabinoxylans (copolymers of** **arabinose and xylose)**	Maintain colon health and resist the absorption of cholesterol in the colon. They are strong prebiotics to maintain gut health by increasing *Lactobacillus* and *Bifidobacterium* population	Maize fibre	Corn fibre gum and xylooligosaccharides Maize arabinoxylans- functional gluten-free bread	(*77*–*79*)
**Protein hydrolysates: peptides**	Antihypertensive, hepatoprotective, anti-inflammatory, increase alcohol metabolism, antimicrobial	Maize gluten meal	Maize bioactive oligopeptide extracted from non-GMO maize and spray dried powder (smart PEP)	(*49*, *60*–*62*, *80*)

This table demonstrates that the maize wet milling by-products are rich in phytochemicals and can be transformed into functional food ingredients or nutraceuticals. Functional food ingredients/nutraceutical supplements are available from various expensive sources, such as the carotenoids from marigold flowers, policosanols from sugarcane or beeswax. However, the by-products of the maize wet milling process are an alternative source for manufacturing nutraceuticals and functional food ingredients.

## CONCLUSIONS

Wet milling of maize yields 60−70% starch with more than 30% by-products (maize germ, gluten and fibre). These by-products are currently used as low-quality feed for poultry, farm animals, pigs and fish. Research studies confirm that they are rich in phytochemicals, mainly tocols, phytosterols, phospholipids, carotenoids, phenolic compounds and arabinoxylans (dietary fibre). Maize germ is rich in tocols, maize fibre is rich in phytosterols and dietary fibre, whereas maize gluten (protein) is rich in carotenoids and proteins. Some functional foods produced from by-products of maize wet milling include germ oil, fibre oil and gum, and protein hydrolysates and peptides from maize gluten. These functional foods are reported to have beneficial health effects like cholesterol-lowering, cardioprotective, hepatoprotective, anticarcinogenic and prebiotic properties. The by-products can be upcycled to high-quality nutraceutical sources. However, more scientific information about the phytochemical content of maize wet milling by-products is required. Sufficient research is also needed on the effects of technological interventions such as thermal, physical and enzymatic treatments on the nutraceutical quality of maize wet milling by-products to enable the development of functional food ingredients or dietary supplements. Overall, these by-products can establish a lucrative platform for industrial maize wet milling, the bioethanol industry, and farmers to economically transform the agricultural sector.

## References

[1] Sandhu KS Singh N Malhi NS. Some properties of corn grains and their flours I: Physicochemical, functional and chapati-making properties of flours. Food Chem. 2007;101(3):938–46. 10.1016/j.foodchem.2006.02.040

[2] Foreign Agricultural Service (FAS). Grain: World markets and trade. Washington, DC, USA: United States Department of Agriculture (USDA) Economics, Statistics and Market Information System; 2021. Available from: https://usda.library.cornell.edu/concern/publications/zs25x844t?locale=en.

[3] Ranum P Peña-Rosas JP Garcia-Casal MN. Global maize production, utilization, and consumption. Ann N Y Acad Sci. 2014;1312(1):105–12. 10.1111/nyas.12396 24650320

[4] India maize scenario. PAU Campus, Ludhiana, Punjab, India: ICAR - Indian Institute of Maize research; 2021. Available from: https://iimr.icar.gov.in/india-maze-scenario/.

[5] Wronkowska M. Wet-milling of cereals. J Food Process Preserv. 2016;40(3):572–80. 10.1111/jfpp.12626

[6] Rausch KD, Eckhoff SR. Maize: Wet milling. In: Wrigley C, Corke H, Seetharaman, Faubion J, editors. Encyclopedia of food grains. Amsterdam, The Netherlands: Elsevier Inc; 2016. pp. 467–81. 10.1016/B978-0-12-394437-5.00239-4 10.1016/B978-0-12-394437-5.00239-4

[7] Rose DJ Inglett GE Liu SX. Utilisation of corn (*Zea mays*) bran and corn fiber in the production of food components. J Sci Food Agric. 2010;90(6):915–24. 10.1002/jsfa.3915 20355130

[8] Galanakis CM. Sustainable recovery and reutilization of cereal processing by-products. Amsterdam, The Netherlands: Elsevier Inc; 2018. 10.1016/B978-0-08-102162-0.01001-7 10.1016/B978-0-08-102162-0.01001-7

[9] Chaudhary DP, Kumar S, Langyan S, editors. Maize: Nutrition dynamics and novel uses. New Delhi, India: Springer India; 2014. 10.1007/978-81-322-1623-0 10.1007/978-81-322-1623-0

[10] Loy DD, Lundy EL. Nutritional properties and feeding value of corn and its coproducts. In: Serna-Saldivar SO, editor. Corn: Chemistry and technology. Amsterdam, The Netherlands: Elsevier Inc; 2019. pp. 633–59. 10.1016/B978-0-12-811971-6.00023-1 10.1016/B978-0-12-811971-6.00023-1

[11] Tekchandani HK Dias FF Mehta D. Maize wet milling co-products as feed additives: Perspectives and opportunities. J Sci Ind Res (India). 1999;58(2):83–8.

[12] Kumar D, Singh V. Bioethanol production from corn. In: Serna-Saldivar SO, editor. Corn: Chemistry and technology. Amsterdam, The Netherlands: Elsevier Inc; 2019. pp. 615–31. 10.1016/B978-0-12-811971-6.00022-X 10.1016/B978-0-12-811971-6.00022-X

[13] From niche to nation. Ethanol industry outlook 2006. Washington, DC, USA: Renewable Fuels Association; 2006. pp. 4–21. Available from: http://www.cornlp.com/Adobe/outlook2006.pdf.

[14] Tan SL Morrison WR. The distribution of lipids in the germ, endosperm, pericarp and tip cap of amylomaize, LG-11 hybrid maize and waxy maize. J Am Oil Chem Soc. 1979;56(4):531–5. 10.1007/BF02680196

[15] Acosta-Estrada BA, Gutiérrez-Uribe JA, Serna-Saldivar SO. Minor constituents and phytochemicals of the kernel. In: Serna-Saldivar SO, editor. Corn: Chemistry and technology. Amsterdam, The Netherlands: Elsevier Inc; 2019. pp. 369–403. 10.1016/B978-0-12-811971-6.00014-0 10.1016/B978-0-12-811971-6.00014-0

[16] Weber EJ. Carotenoids and tocols of corn grain determined by HPLC. J Am Oil Chem Soc. 1987;64(8):1129–34. 10.1007/BF02612988

[17] Ko SN Kim CJ Kim H Kim CT Chung SH Tae BS Tocol levels in milling fractions of some cereal grains and soybean. J Am Oil Chem Soc. 2003;80(6):585–9. 10.1007/s11746-003-0742-9

[18] Zheng L Jin J Huang J Wang Y Korma SA Wang X Effects of heat pretreatment of wet-milled corn germ on the physicochemical properties of oil. J Food Sci Technol. 2018;55(8):3154–62. 10.1007/s13197-018-3243-6 30065426 PMC6046002

[19] Grams GW Blessin CW Inglett GE. Distribution of tocopherols in wet- and dry-milled corn products. Cereal Chem. 1971;48(4):356–9.

[20] Singh V Moreau RA Hicks KB. Yield and phytosterol composition of oil extracted from grain sorghum and its wet-milled fractions. Cereal Chem J. 2003;80(2):126–9. 10.1094/CCHEM.2003.80.2.126

[21] Aydeniz Güneşer B Yılmaz E Ok S. Cold pressed versus refined winterized corn oils: Quality, composition and aroma. Grasas Aceites. 2017;68(2):1168162. 10.3989/gya.1168162

[22] Moreau RA Hicks KB. Reinvestigation of the effect of heat pretreatment of corn fiber and corn germ on the levels of extractable tocopherols and tocotrienols. J Agric Food Chem. 2006;54(21):8093–102. 10.1021/jf061422g 17032015

[23] Inglett GE Blessin CW. Food applications of corn germ protein products. J Am Oil Chem Soc. 1979;56(3):479–80. 10.1007/BF02671550

[24] Liu H Liu T Fan H Gou M Li G Ren H Corn lecithin for injection from deoiled corn germ: Extraction, composition, and emulsifying properties. Eur J Lipid Sci Technol. 2018;120(3):1700288. 10.1002/ejlt.201700288

[25] Yadav MP Johnston DB Hotchkiss AT Hicks KB. Corn fiber gum: A potential gum arabic replacer for beverage flavor emulsification. Food Hydrocoll. 2007;21(7):1022–30. 10.1016/j.foodhyd.2006.07.009

[26] Singh V Johnston DB Moreau RA Hicks KB Dien BS Bothast RJ. Pretreatment of wet-milled corn fiber to improve recovery of corn fiber oil and phytosterols. Cereal Chem J. 2003;80(2):118–22. 10.1094/CCHEM.2003.80.2.118

[27] Yadav MP Moreau RA Hicks KB. Phenolic acids, lipids, and proteins associated with purified corn fiber arabinoxylans. J Agric Food Chem. 2007;55(3):943–7. 10.1021/jf0624493 17263497

[28] Yadav MP Moreau RA Hotchkiss AT Hicks KB. A new corn fiber gum polysaccharide isolation process that preserves functional components. Carbohydr Polym. 2012;87(2):1169–75. 10.1016/j.carbpol.2011.08.092

[29] Hamaker BR, Tuncil YE, Shen X. Carbohydrates of the kernel. In: Serna-Saldívar SO, editor. Corn: Chemistry and technology. Amsterdam, The Netherlands: Elsevier Inc; 2019. pp. 305–18. 10.1016/B978-0-12-811971-6.00011-5 10.1016/B978-0-12-811971-6.00011-5

[30] Guevara MA Bauer LL Abbas CA Beery KE Holzgraefe DP Cecava MJ Chemical composition, *in vitro* fermentation characteristics, and *in vivo* digestibility responses by dogs to select corn fibers. J Agric Food Chem. 2008;56(5):1619–26. 10.1021/jf073073b 18275146

[31] Vázquez M Alonso J Domínguez H Parajó J. Xylooligosaccharides: Manufacture and applications. Trends Food Sci Technol. 2000;11(11):387–93. 10.1016/S0924-2244(01)00031-0

[32] Chung YC Hsu CK Ko CY Chan YC. Dietary intake of xylooligosaccharides improves the intestinal microbiota, fecal moisture, and pH value in the elderly. Nutr Res. 2007;27(12):756–61. 10.1016/j.nutres.2007.09.014

[33] Ou S Kwok KC. Ferulic acid: Pharmaceutical functions, preparation and applications in foods. J Sci Food Agric. 2004;84(11):1261–9. 10.1002/jsfa.1873

[34] Saulnier L Marot C Chanliaud E Thibault JF. Cell wall polysaccharide interactions in maize bran. Carbohydr Polym. 1995;26(4):279–87. 10.1016/0144-8617(95)00020-8

[35] Saulnier L Marot C Elgorriaga M Bonnin E Thibault JF. Thermal and enzymatic treatments for the release of free ferulic acid from maize bran. Carbohydr Polym. 2001;45(3):269–75. 10.1016/S0144-8617(00)00259-9

[36] Winkelhausen E Kuzmanova S. Microbial conversion of d-xylose to xylitol. J Ferment Bioeng. 1998;86(1):1–14. 10.1016/S0922-338X(98)80026-3

[37] Rao RS Jyothi CP Prakasham RS Sarma PN Rao LV. Xylitol production from corn fiber and sugarcane bagasse hydrolysates by *Candida tropicalis.* Bioresour Technol. 2006;97(15):1974–8. 10.1016/j.biortech.2005.08.015 16242318

[38] Buhner J Agblevor FA. Effect of detoxification of dilute-acid corn fiber hydrolysate on xylitol production. Appl Biochem Biotechnol. 2004;119(1):13–30. 10.1385/ABAB:119:1:13 15496725

[39] Buranov AU Mazza G. Extraction and purification of ferulic acid from flax shives, wheat and corn bran by alkaline hydrolysis and pressurised solvents. Food Chem. 2009;115(4):1542–8. 10.1016/j.foodchem.2009.01.059

[40] Lesage-Meessen L Lomascolo A Bonnin E Thibault JF Buleon A Roller M A Biotechnological process involving filamentous *Fungi* to produce natural crystalline vanillin from maize bran. Appl Biochem Biotechnol. 2002;102-103(1–6):141–53. 10.1385/ABAB:102-103:1-6:141 12396118

[41] Moreau RA, Singh V, Powell MJ, Hicks KB. Corn kernel oil and corn fiber oil. In: Moreau RA, Kamal-Eldin A, editors. Gourmet and health-promoting specialty oils. Amsterdam, The Netherlands: Elsevier Inc; 2009. pp. 409–31. 10.1016/B978-1-893997-97-4.50021-8 10.1016/B978-1-893997-97-4.50021-8

[42] Moreau RA Lampi AM Hicks KB. Fatty acid, phytosterol, and polyamine conjugate profiles of edible oils extracted from corn germ, corn fiber, and corn kernels. J Am Oil Chem Soc. 2009;86(12):1209–14. 10.1007/s11746-009-1456-6

[43] Ramjiganesh T Roy S Freake HC McIntyre JC Fernandez ML. Corn fiber oil lowers plasma cholesterol by altering hepatic cholesterol metabolism and up-regulating LDL receptors in guinea pigs. J Nutr. 2002;132(3):335–40. 10.1093/jn/132.3.335 11880551

[44] Wilson TA DeSimone AP Romano CA Nicolosi RJ. Corn fiber oil lowers plasma cholesterol levels and increases cholesterol excretion greater than corn oil and similar to diets containing soy sterols and soy stanols in hamsters. J Nutr Biochem. 2000;11(9):443–9. 10.1016/S0955-2863(00)00103-0 11091099

[45] Leguizamón C Weller CL Schlegel VL Carr TP. Plant sterol and policosanol characterization of hexane extracts from grain sorghum, corn and their DDGS. J Am Oil Chem Soc. 2009;86(7):707–16. 10.1007/s11746-009-1398-z

[46] Harrabi S Boukhchina S Mayer PM Kallel H. Policosanol distribution and accumulation in developing corn kernels. Food Chem. 2009;115(3):918–23. 10.1016/j.foodchem.2008.12.098

[47] Nuss ET Tanumihardjo SA. Maize: A paramount staple crop in the context of global nutrition. Compr Rev Food Sci Food Saf. 2010;9(4):417–36. 10.1111/j.1541-4337.2010.00117.x 33467836

[48] Li X Han L Chen L. *In vitro* antioxidant activity of protein hydrolysates prepared from corn gluten meal. J Sci Food Agric. 2008;88(9):1660–6. 10.1002/jsfa.3264

[49] Li G, Liu W, Wang Y, Jia F, Wang Y, Ma Y, et al. Functions and applications of bioactive peptides from corn gluten meal. In: Toldrá F, editor. Advances in food and nutrition research. Amsterdam, The Netherlands: Elsevier Inc; 2019. pp. 1–41. 10.1016/bs.afnr.2018.07.001 10.1016/bs.afnr.2018.07.001 30678813

[50] Heuzé V, Tran G, Sauvant D, Renaudeau D. Lessire. M, Lebas F. Corn gluten meal. St. Remy de Provence, France: Feedipedia, a programme by INRAE, CIRAD, AFZ and FAO; 2018. Available from: https://www.feedipedia.org/node/715.

[51] Eisenhauer B Natoli S Liew G Flood VM. Lutein and zeaxanthin—Food sources, bioavailability and dietary variety in age-related macular degeneration protection. Nutrients. 2017;9(2):120. 10.3390/nu9020120 28208784 PMC5331551

[52] Saini RK Keum YS. Carotenoid extraction methods: A review of recent developments. Food Chem. 2018;240:90–103. 10.1016/j.foodchem.2017.07.099 28946359

[53] Abdel-Aal SM Akhtar H Zaheer K Ali R. Dietary sources of lutein and zeaxanthin carotenoids and their role in eye health. Nutrients. 2013;5(4):1169–85. 10.3390/nu5041169 23571649 PMC3705341

[54] Moreno FS Toledo LP de Conti A Heidor R Jordão A Vannucchi H Lutein presents suppressing but not blocking chemopreventive activity during diethylnitrosamine-induced hepatocarcinogenesis and this involves inhibition of DNA damage. Chem Biol Interact. 2007;168(3):221–8. 10.1016/j.cbi.2007.04.011 17559825

[55] Zhou K Sun S Canning C. Production and functional characterisation of antioxidative hydrolysates from corn protein *via* enzymatic hydrolysis and ultrafiltration. Food Chem. 2012;135(3):1192–7. 10.1016/j.foodchem.2012.05.063 22953842

[56] Schlimme E, Meisel H. Bioactive peptides derived from milk proteins. Structural, physiological and analytical aspects. Food/Nahrung. 1995;39(1):1–20. 10.1002/food.19950390102 10.1002/food.19950390102 7898574

[57] Wang XJ Zheng XQ Kopparapu NK Cong WS Deng YP Sun XJ Purification and evaluation of a novel antioxidant peptide from corn protein hydrolysate. Process Biochem. 2014;49(9):1562–9. 10.1016/j.procbio.2014.05.014

[58] Lin F Chen L Liang R Zhang Z Wang J Cai M Pilot-scale production of low molecular weight peptides from corn wet milling by-products and the antihypertensive effects *in vivo* and *in vitro.* Food Chem. 2011;124(3):801–7. 10.1016/j.foodchem.2010.06.099

[59] Zhu B He H Hou T. A comprehensive review of corn protein-derived bioactive peptides: Production, characterization, bioactivities, and transport pathways. Compr Rev Food Sci Food Saf. 2019;18(1):329–45. 10.1111/1541-4337.12411 33337020

[60] Guo H Sun J He H Yu GC Du J. Antihepatotoxic effect of corn peptides against Bacillus Calmette-Guerin/lipopolysaccharide-induced liver injury in mice. Food Chem Toxicol. 2009;47(10):2431–5. 10.1016/j.fct.2009.06.041 19577609

[61] Ma ZL Zhang WJ Yu GC He H Zhang Y. The primary structure identification of a corn peptide facilitating alcohol metabolism by HPLC–MS/MS. Peptides. 2012;37(1):138–43. 10.1016/j.peptides.2012.07.004 22789607

[62] Huang WH Sun J He H Dong HW Li JT. Antihypertensive effect of corn peptides, produced by a continuous production in enzymatic membrane reactor, in spontaneously hypertensive rats. Food Chem. 2011;128(4):968–73. 10.1016/j.foodchem.2011.03.127

[63] García-Olmedo F Molina A Alamillo JM Rodríguez-Palenzuéla P. Plant defense peptides. Biopolymers. 1998;47(6):479–91. 10.1002/(SICI)1097-0282(1998)47:6<479::AID-BIP6>3.0.CO;2-K 10333739

[64] Epand RM Vogel HJ. Diversity of antimicrobial peptides and their mechanisms of action. Biochim Biophys Acta. 1999;1462(1–2):11–28. 10.1016/S0005-2736(99)00198-4 10590300

[65] Zeisel SH. Regulation of “Nutraceuticals”. Science. 1999;285(5435):1853–5. 10.1126/science.285.5435.1853 10515789

[66] Heuzé V, Tran G, Lebas F. Maize germ meal and maize germ. St. Remy de Provence, France: Feedipedia, a programme by INRAE, CIRAD, AFZ and FAO; 2015. pp. 1–6. Available from: https://www.feedipedia.org/node/716.

[67] Hespell RB. Extraction and characterization of hemicellulose from the corn fiber produced by corn wet-milling processes. J Agric Food Chem. 1998;46(7):2615–9. 10.1021/jf971040y

[68] Grohmann K Bothast RJ. Saccharification of corn fibre by combined treatment with dilute sulphuric acid and enzymes. Process Biochem. 1997;32(5):405–15. 10.1016/S0032-9592(96)00095-7

[69] Maize in human nutrition. Rome, Italy: Food and Agriculture Organization (FAO) of the United Nations; 1992. Available from: https://www.fao.org/3/t0395e/T0395E00.htm#Contents.

[70] Moreau RA Powell MJ Hicks KB. Extraction and quantitative analysis of oil from commercial corn fiber. J Agric Food Chem. 1996;44(8):2149–54. 10.1021/jf950743h

[71] Barrera-Arellano D, Badan-Ribeiro AP, Serna-Saldivar SO. Corn oil: Composition, processing, and utilization. In: Serna-Saldívar SO, editor. Corn: Chemistry and technology. Amsterdam, The Netherlands: Elsevier Inc; 2019. pp. 593–613. 10.1016/B978-0-12-811971-6.00021-8 10.1016/B978-0-12-811971-6.00021-8

[72] Ribiero K de O Garcia MC Oliviera AR. Soares Junior MS, Caliri M. Characterization and proposal of potential use in foods of co-products from waxy maize wet milling. Food Sci Technol. 2019;39(2):315–20. 10.1590/fst.26817

[73] Siyuan S Tong L Liu RH. Corn phytochemicals and their health benefits. Food Sci Hum Wellness. 2018;7(3):185–95. 10.1016/j.fshw.2018.09.003

[74] Jiang Y Wang T. Phytosterols in cereal by-products. J Am Oil Chem Soc. 2005;82(6):439–44. 10.1007/s11746-005-1090-5

[75] Wang T, White PJ. Lipids of the Kernel. In: Serna-Saldívar SO, editor. Corn: Chemistry and technology. Amsterdam, The Netherlands: Elsevier Inc; 2019. pp. 337–68. 10.1016/B978-0-12-811971-6.00013-9 10.1016/B978-0-12-811971-6.00013-9

[76] Serna-Saldívar SO. Role of cereals in human nutrition and health. In: Cereal grains properties, processing, and nutritional attributes. Boca Raton, FL, USA: CRC Press; 2010. pp. 606–16.

[77] Ogawa K Takeuchi M Nakamura N. Immunological effects of partially hydrolyzed arabinoxylan from corn husk in mice. Biosci Biotechnol Biochem. 2005;69(1):19–25. 10.1271/bbb.69.19 15665462

[78] Niño-Medina G Carvajal-Millán E Rascon-Chu A Marquez-Escalante JA Guerrero V Salas-Muñoz E. Feruloylated arabinoxylans and arabinoxylan gels: Structure, sources and applications. Phytochem Rev. 2010;9(1):111–20. 10.1007/s11101-009-9147-3

[79] Bastos R, Coelho E, Coimbra MA. Arabinoxylans from cereal by-products: Insight into strutural features,recovery, and applications. In: Galanakis CM, editor. Sustainable recovery and reutilization of cereal processing by-products. Amsterdam, The Netherlands: Elsevier Inc; 2018. pp. 227–51. 10.1016/B978-0-08-102162-0.00008-3 10.1016/B978-0-08-102162-0.00008-3

[80] Zhang F Zhang J Li Y. Corn oligopeptides protect against early alcoholic liver injury in rats. Food Chem Toxicol. 2012;50(6):2149–54. 10.1016/j.fct.2012.03.083 22504530

